# Neurodevelopmental delay among children under the age of three years at immunization clinics in Lagos State, Nigeria – Preliminary report

**DOI:** 10.1038/srep25175

**Published:** 2016-04-29

**Authors:** Muideen O. Bakare, Mashudat A. Bello-Mojeed, Kerim M. Munir, Oluwayemi C. Ogun, Julian Eaton

**Affiliations:** 1 Child and Adolescent Unit, Federal Neuropsychiatric Hospital, Upper Chime, New Haven, Enugu, Enugu State, Nigeria; 2 Child and Adolescent Unit, Federal Neuropsychiatric Hospital, Yaba, Lagos, Nigeria; 3 University Center for Excellence in Developmental Disabilities (UCEDD), Division of Developmental Medicine, Children’s Hospital and Harvard Medical School, Boston, Massachusetts, USA; 4 CBM, West Africa Regional Office, Villa B-86, Rue des Mercuriales, Residence du Benin, BP 13489 – Lomé, Togo; 5 Childhood Neuropsychiatric Disorders Initiatives (CNDI), E46 Goshen Estate, Premier Layout, Enugu, Enugu State, Nigeria

## Abstract

Late diagnosis and interventions characterize childhood neurodevelopmental disorders in Sub-Saharan Africa. This has negatively impacted on the prognosis of the children with neurodevelopmental disorders. This study examined the prevalence and pattern of neurodevelopmental delays among children under the age of 3 years attending immunization clinics in Lagos State, Nigeria and also affords opportunity of early follow-up and interventions, which had been documented to improve prognosis. The study involved two stage assessments; which consisted of first phase screening of the children for neurodevelopmental delays in immunization clinics at primary healthcare centers Lagos State, Nigeria and second phase which consists of definitive clinical evaluation and follow-up interventions for children screened positive for neurodevelopmental delays. Twenty seven (0.9%) of a total of 3,011 children under the age of 3 years were screened positive for neurodevelopmental delays and subsequently undergoing clinical evaluation and follow-up interventions. Preliminary working diagnoses among these children include cerebral palsy, autism spectrum disorder trait, nutritional deficiency, Down syndrome and Non-specific neurodevelopmental delay with co-morbid seizure disorder accounting for 33.3%, 14.8%, 18.5%, 7.4% and 25.9% respectively. This is a preliminary report that would be followed up with information on medium and long term intervention phase.

Neurodevelopmental disorders (NDD) are group of disorders arising from impairments in the developing brain and/or the central nervous system. They are considered neurodevelopmental in that by definition they originate during the developmental period, that is, during the prenatal, ante-natal, post-natal, infancy and early childhood periods^1^. The disorders have varying degrees of associated burden on children, their families and their communities and almost always require multi-faceted services to address special educational, health care, social inclusion and rehabilitation needs^1^.

The NDD include intellectual developmental disorders with known genetic or metabolic etiologies, traumatic or congenital brain injuries including conditions such as cerebral palsy, as well as prenatal exposures such as fetal alcohol syndrome, and disorders of social relatedness such as autism spectrum disorders^1^.

With the declining under-five mortality in recent years, report suggests a possible future high prevalence paradigm shift from communicable diseases to childhood neurodevelopmental disorders in Sub-Saharan Africa as more children survive beyond the age of five years^1^.

The study is part of a project titled, “Early Identification and Intervention for Childhood Neurodevelopmental Disorders among Children under the age of 3 years in Lagos State, Nigeria”. This project incorporates screening and interventional follow-up for neurodevelopmental disorders among children under the age of three years.

Prior to now, data are not available regarding the prevalence and pattern of neurodevelopmental disorders among young children under the age of 3 years in Nigeria and other sub-Saharan African countries. This is often as a result of late diagnosis and interventions that characterized neurodevelopmental disorders in this region, with earliest diagnoses often made at school age between the ages of 6 and 8 years, leading to late interventions for these children^2^. Details about factors that may be responsible for the late diagnosis in sub-Saharan Africa are contained in a previous review^2^.

Having baseline data about prevalence and pattern of neurodevelopmental delay in this age group would help healthcare and educational services planning for such children, who may have special education needs.

The Federal Government of Nigeria in 1999 introduced the National Program on Immunization (NPI) to replace the existing Expanded Program on Immunization (EPI) that was initiated in 1979. The NPI was established with a key focus to provide support to the implementation of the State and Local Government Area (LGA) immunization programs^3^.

Immunization clinics at the primary healthcare centers constitute major clinical source of convergence for children under the age of 3 years and their mothers. The immunization clinic environment also affords the opportunity for early screening for neurodevelopmental delay among these children in a less stigmatizing setting.

This study assessed the prevalence and pattern of neurodevelopmental delays among children under the age of 3 years attending immunization clinics in primary health care centers in two randomly selected Local Government Areas (LGAs) of the twenty LGAs in Lagos State, Nigeria.

This is aimed at providing preliminary baseline data on prevalence and pattern of neurodevelopmental delays among children under the age of three years in Nigeria and also to provide early interventional follow up for these children, which had been documented to improve prognosis^1^.

The study is a preliminary report of the project titled, “Early Diagnosis and Interventions for Childhood Neurodevelopmental Disorders (NDD) among Children under the Age of 3 Years in Lagos State, Nigeria” supported by Grand Challenges Canada (GCC).

## Methods

### Location

The locations of the study were immunization clinics of primary healthcare centers in two of twenty Local Government Areas (LGAs) in Lagos State, South West Nigeria. Lagos State is the second most populous State in Nigeria after Kano out the 36 States and Federal Capital Territory (FCT) in Nigeria. It is sub-divided into 20 Local Government Areas (LGAs). Each of the LGA is involved in National Program on Immunization (NPI) at Primary Health Care (PHC) level^1^.

## Materials

### Sociodemographic Questionnaire

This was used to obtain basic demographic information of the mothers and children attending the immunization clinics.

### WHO Child Growth Standard Charts

Growth charts are visible displays of a child’s physical growth and development^4^. WHO standard growth charts have two reference curves for both sexes in relation to weight for age, length for age and head circumference for age. Normal variations are assumed to include two standard deviations above and below the mean, that is, 3rd and 97^th^ percentile. Recordings below the 3rd percentile would be said to be an indication of delayed growth. For this study, the weight for age, length for age and Head Circumference for age charts for both sexes were used. WHO Child Growth Standard Chart was used in this study to assess length for age, weight for age, and head circumference for age for each child studied, with the aim of further assessment for problem of malnutrition in a child with recording below 3^rd^ percentile in any of the measured parameters.

### Infant Development Inventory (IDI)

The IDI has introductory part that asked the parents to describe their child generally and a second part that assessed the child development specifically in the areas of; *Social*; *Self-Help*; *Gross Motor*; *Fine Motor* and Language, which help to estimate the mental age of the child^5^. Further details about this Instrument can be found in the url link in the reference. This was used to screen for parental developmental concern in children within the age range of 0 to 18 Months.

### Child Development Review (CDR)

The CDR has introductory part that asked the parents to describe their child generally and a second part that assessed the child development specifically in the areas of; *Social; Self-Help; Gross Motor; Fine Motor* and *Language*, which help to estimate the mental age of the child^6^. The CDR also contained additional information or checklist of additional problems that the child may be experiencing. Further details about this Instrument can be found in the url link in the reference. This was used to screen for parental developmental concern in children above 18 Months of age.

### CDC Mile Stone Moments (CDCMM)

CDC Mile Stone Moments assessed child development specifically in the areas of; *Social/Emotional; Language/Communication; Cognitive; Movement/Physical Development*^7^. Further details about this Instrument can be found in the url link in the reference. This was used to screen for parental developmental concern in children within the age range of 0 to 36 Months.

## Ethical Consideration

The objective of the study was explained to the mothers bringing their children to the immunization clinics and their inform consent obtained before the screening and anthropometric measurements for the children. The ethical approval for this study was obtained from the Institutional Review Board (IRB) of Federal Neuropsychiatric Hospital, Yaba, Lagos, Lagos State Nigeria. The methods for this project were carried out in accordance with the approved guidelines.

## Procedure

The diagnostic procedure was in two phases consisting of screening and clinical evaluation:

### First Phase

The first phase consisted of screening of the children for neurodevelopmental delays with IDI, CDR and CDCMM through parental report regarding the achievement of different milestones by the individual child. During this phase, anthropometric measurements of weight, length and head circumference were also done for individual child seen at immunization clinics. A total of 3,011 mothers (98.2%) out of the 3,065 mothers approached over a two year period of the project consented to screening and anthropometric measurement for their children. The children were screened consecutively as they attended immunization clinics. The first 1,500 children were screened with either IDI^5^ and CDCMM^7^ or CDR^6^ and CDCMM^7^, depending on the age of a particular child at the time of screening. Based on the first 1,500 children screened, it was observed that CDCMM showed 100% sensitivity against IDI and CDR in detecting children with neurodevelopmental delays based on the parents’ report. The rest of the children (1,511) were screened with only CDC Milestone Moments^7^, because of the commercial implication of the other two screening tools (IDI and CDR). Out of a total of 3,011 children screened for neurodevelopmental delay, 27 (0.9%) screened positive for neurodevelopmental delay and were qualified for second phase which was definitive clinical evaluation and follow-up.

### Second Phase

The second phase of clinical evaluation and follow-up interventions, presently ongoing at the Child and Adolescent Center of Federal Neuropsychiatric Hospital, Yaba, Lagos is examining the following parameters for each individual child screened positive for neurodevelopmental delay: developmental trajectory/developmental milestone; cognitive trajectory/education needs assessment; adaptive functioning; biomedical profile; co-morbid physical health problems; nutritional components and definitive diagnosis. As the present period represents preliminary follow-up period, detail information about many of these parameters are presently inconclusive. For example, information about cognitive trajectory and adaptive functioning is presently not available, as assessment of these parameters is deferred till school age, which is about six (6) years in this environment. Assessment of cognitive trajectory and adaptive functioning would follow the methods highlighted in a previous publication in this environment^8^, in view of lack of standardized culturally adaptive tools. However, we are able to report at this time pattern of preliminary diagnoses we are working with among the 27 children screened positive for neurodevelopmental delays. The preliminary diagnoses were made following diagnostic criteria specified in the International Classification of Diseases, tenth Edition (ICD-10). The average follow-up period till date for these children is about three (3) months.

## Results

A total of 3,011 children were screened at the immunization clinics under the primary healthcare centers of two Local Government Areas of Mushin and Oshodi in Lagos State, South West Nigeria. This figure represents 98.2% of total numbers of children and mothers approached to be screened.

Out of the total of 3,011 children screened, there are 1,587 female children (52.7%) and 1,424 male children (47.3%). The age range of the children screened and followed up over the two year period of the project is 3 to 36 months. Mean age was about 6.0 months at the point of first screening. Each child screened was followed up for at least 6 months following the first screening to rule out any fresh sign of neurodevelopmental delay after the first screening.

A total of 27 children (0.9%) were screened positive for neurodevelopmental delay. Out of this total, there are 14 males (1.0%) and 13 females (0.8%), showing that male children are more likely to experience neurodevelopmental delay. However, this difference was not statistically significant (X^2^ = 0.23, df = 1, p = 0.634). The age range of children with neurodevelopmental delay is 3 to 31 months. Their mean age is 12.8 ± 8.9 months, with a median age of 9.0 months. Table 1 showed gender information of children screened positive for neurodevelopmental delay.

### Preliminary working diagnoses pattern

Table 2 showed preliminary working diagnoses pattern of children screened positive for neurodevelopmental delays and other follow up information till date. Cerebral palsy, Autism Spectrum Disorder trait, Nutritional deficiency, Down Syndrome and Non-Specific neurodevelopmental delay with co-morbid seizure disorder accounted for 33.3%, 14.8%, 18.5%, 7.4% and 25.9% of the neurodevelopmental delays observed respectively.

## Discussion

In the first phase of the screening at the immunization clinics, this study adopted the approach of using screening tools to elicit parental concerns about development of their children under the age of three (3) years. This approach combined the two methods of screening with tools and eliciting parental concerns, both of which had been identified as effective ways of early screening for neurodevelopmental and behavioral problems^9^. It has been documented that parental information and concerns are important in early detection of neurodevelopmental problems in affected children^10^,^11^,^12^.

Prevalence of neurodevelopmental delays among children under the age of three years in this study was found to be 0.9%. Cerebral palsy, Autism Spectrum Disorder trait, Nutritional deficiency, Down Syndrome and Non-Specific neurodevelopmental delay with co-morbid seizure disorder accounted for 33.3%, 14.8%, 18.5%, 7.4% and 25.9% of this prevalence respectively.

Male children are more likely to experience neurodevelopmental delay compare to the female children. However, this difference is not statistically significant.

It is difficult to compare these findings with previous studies, because this is probably the first time that neurodevelopmental delay screening would be focusing on young children under the age of three years at immunization clinic setting in a Sub-Saharan African sub-region.

Late diagnosis and interventions have characterized neurodevelopmental disorders in Sub-Saharan African children population despite usual early parental concern about development^2^. As a result of late interventions for children with neurodevelopmental disorders in the region, their optimal functioning is not achieved as depicted by many of them not having access to any form of education and impairment in expressive language ability^2^.

The need to diagnose early neurodevelopmental disorder and thus provide early interventions and follow-up which had been documented to improve prognosis^1^,^2^ informs this study.

Follow-up for these children would continue through fortnight clinical appointments at the tertiary care center, Child and Adolescent Center of Federal Neuropsychiatric Hospital, Yaba, Lagos, Nigeria.

## Limitation

The study provides preliminary data that cannot be generalized as at present. There is need for Government buy-in to produce a nationally representative data that would be useful for healthcare and educational services planning for children with neurodevelopmental disability in the country.

## Conclusions

The findings of this study showed preliminary data about prevalence and pattern of neurodevelopmental delays in children under the age of three years in Nigeria. Expanding this process nationally in Nigeria would afford availability of baseline data that can help in healthcare and educational services planning for this group of children with neurodevelopmental disability. The process would also afford early follow-up and interventions for the children with the ultimate aim of improving their functioning and prognosis.

Future direction should focus on lobbying for and enacting legislations that would promote community inclusion for children and adults with neurodevelopmental disability in Nigeria.

## Additional Information

**How to cite this article**: Bakare, M. O. *et al.* Neurodevelopmental delay among children under the age of three years at immunization clinics in Lagos State, Nigeria – Preliminary report. *Sci. Rep.* **6**, 25175; doi: 10.1038/srep25175 (2016).

## Figures and Tables

**Table 1:** Gender Distribution of children screened positive for developmental delay.

Gender	Children Screened N (%)	Children with Developmental Delay N (%)
Male	1,424 (47.3)	14 (1.0)
Female	1,587 (52.7)	13 (0.8)
Total	3,011 (100)	27 (0.9)

**Table 2:** Prevalence and pattern of preliminary working diagnoses of children screened positive for neuro-developmental delay in immunization clinics in South West Nigeria.

Preliminary Working Diagnosis	Frequency N (%)	Comments on Follow-up	Present Frequency on Follow-up N (%)
Cerebral Palsy ± Seizure Disorder	9 (33.3)	Presently on follow-up at Tertiary Care Center	9 (34.6)
Autism Spectrum Disorder (ASD) Trait	4 (14.8)	Presently on follow-up at Tertiary Care Center	4 (15.4)
Nutritional Deficiency	5 (18.5)	All children improved following 3 Months period of follow-up and nutritional advice to the mothers (achieved at least 3rd percentile weight gain and length on WHO Standard Growth Charts). Still on follow-up	5 (19.2)
Down Syndrome	2 (7.4)	Presently on follow-up at Tertiary Care Center	2 (7.7)
Non Specific developmental delay with co-morbid Seizure Disorder	7 (25.9)	One (1) of these children is lost to death from Aspiration following Seizure Episode (Parents Report). The rest are still on follow-up	6 (23.1)
Total	27 (100)		26 (100)
